# Full-length transcriptome analysis of multiple organs and identification of adaptive genes and pathways in *Mikania micrantha*

**DOI:** 10.1038/s41598-022-07198-0

**Published:** 2022-02-28

**Authors:** Xiaoxian Ruan, Zhen Wang, Yingjuan Su, Ting Wang

**Affiliations:** 1 School of Life Sciences, Sun Yat-sen University, Guangzhou, 510275 China; 2 Research Institute of Sun Yat-sen University in Shenzhen, Shenzhen, 518057 China; 3 College of Life Sciences, South China Agricultural University, Guangzhou, 510641 China

**Keywords:** Plant sciences, Ecology

## Abstract

*Mikania micrantha* is a notorious invasive weed that has caused huge economic loss and negative ecological consequences in invaded areas. This species can adapt well to invasive environments with various stress factors. The identification of gene families and functional pathways related to environmental adaptability is lack in *M. micrantha* at the multi-organ full-length transcriptome level. In this study, we sequenced the transcriptomes of five *M. micrantha* organs using PacBio single-molecule real-time sequencing and Illumina RNA sequencing technologies. Based on the transcriptome data, full-length transcripts were captured and gene expression patterns among the five organs were analyzed. KEGG enrichment analysis of genes with higher expression indicated their special roles in environmental stress response and adversity adaptation in the various five organs. The gene families and pathways related to biotic and abiotic factors, including terpene synthases, glutathione S-transferases, antioxidant defense system, and terpenoid biosynthesis pathway, were characterized. The expression levels of most differentially expressed genes in the antioxidant defense system and terpenoid biosynthesis pathway were higher in root, stem, and leaf than in the other two organs, suggesting that root, stem, and leaf have strong ability to respond to adverse stresses and form the important organs of terpenoid synthesis and accumulation. Additionally, a large number of transcription factors and alternative splicing events were predicted. This study provides a comprehensive transcriptome resource for *M. micrantha*, and our findings facilitate further research on the adaptive evolution and functional genomics of this species.

## Introduction

*Mikania micrantha* (Asteraceae), a perennial creeping herb or semi-woody vine originating from Central and south America, is ranked among the top ten worst weeds in the world^1^. The rapid expansion of this species throughout Pacific Islands and Southeast and South Asia regions, including south China, has caused huge economic loss and negative ecological consequences^2^. *Mikania micrantha* is widely found in agricultural and forest lands, open disturbed areas, and various climatic niches and biomes^2,3^. Strong adaptability of *M. micrantha* is its remarkable characteristic, and the reason behind it has become the focus of attention. High photosynthetic capacity is a representative feature of *M. micrantha*, which provides sufficient energy supply for rapid vegetative propagation and is conducive to invade ecosystems with different light intensities^4^. Strong allelopathic effect is another notable trait of *M. micrantha*. Its essential oil contains abundant terpenoids, especially monoterpenes and sesquiterpenes, which inhibit the growth of other plants and have a significant deterrent effect on oviposition of other insects^5,6^. In addition to high photosynthetic capacity and strong allelopathy, *M. micrantha* can adapt well to invasive environments with various abiotic factors. For example, *M. micrantha* can grow in various soil types, aquatic habitats, high salt conditions, and dry and cold areas^7–9^. These findings suggest that strong adaptability to environmental changes promotes the spread and invasion of *M. micrantha*. Therefore, it is necessary and important to study how *M. micrantha* adapts to the invasive environment for its invasion control.

To cope up with adverse environmental conditions, gene families related to stress, including terpene synthases (TPSs) and glutathione S-transferases (GSTs), have been induced and accumulated to protect invasive plants from stress damage and enhance their environmental adaptability. For example, the up-regulation of TPSs and GSTs in invasive plants *Eupatorium adenophorum*, *Solanum elaeagnifolium*, and *Parthenium hysterophorus* was conducive to enhancing tolerance to different light conditions, wounding stress, drought, and high-salinity environment^10–12^. In addition to gene families, some important functional pathways in invasive plants have also been shown to play roles in responding to stress stimuli. Stress stimuli are usually accompanied by an increase in the production of toxic reactive oxygen species (ROS). Excessive ROS may lead to oxidative stress, disrupt the normal metabolism, and affect plant growth and development^13^. Invasive plants have well-developed antioxidant defense systems to counter the deleterious effects of ROS^14^. Furthermore, terpenoids and their synthetic pathways (cytosolic mevalonate (MVA) and plastidial methylerythritol phosphate (MEP) pathway) are important secondary metabolites and functional pathways for plants to respond to various abiotic and biotic stresses^15^. The systematic analysis of these gene families and functional pathways can help us better understand the environmental adaptability of *M. micrantha*.

Transcriptome analysis is a cost-effective approach from which researchers can analyze gene expression patterns in specific organs and screen important gene families and functional pathways. Three previous studies using next-generation sequencing technology characterized the transcriptomes of *M. micrantha*'s leaf, root and leaf mixtures, and young seedlings^16–18^. However, most of the assembled unigenes were not full-length transcript sequences, and these three studies did not generate gene expression profiles of the specific organs. In particular, the lack of transcriptional expression profiles of flower and seed may lead to the omission of potential genes and metabolic pathways related to environmental adaptation^19^. In addition, short reads generated by next-generation sequencing technology lead to difficulties with the explorations of alternative splicing (AS) events, and make the annotation of transcriptomes incomplete and error-prone. With the advancement of sequencing technology, PacBio single-molecule real-time (SMRT) sequencing can generate a single long read containing the entire transcript sequence without assembly^20^, helping overcome the limitations of next-generation sequencing technology. So far, PacBio SMRT sequencing has been successfully utilized to explore the adaptive mechanism of certain invasive plants^21^.

Here, we aimed to provide a comprehensive transcriptomic profile in different *M. micrantha* organs, namely the root, stem, leaf, flower, and seed, using both PacBio SMRT and Illumina RNA-Seq technology. Our objectives were to analyze the gene expression patterns among the five organs and to identify candidate genes and functional pathways related to abiotic and biotic stress factors. This work is the first comprehensive report on the full-length transcriptome of multiple organs of *M. micrantha* and it also provides valuable molecular resources for future research on the adaptation mechanisms and functional genomics of *M. micrantha*.

## Results

### The full-length sequences of PacBio SMRT sequencing

**Figure 1:**
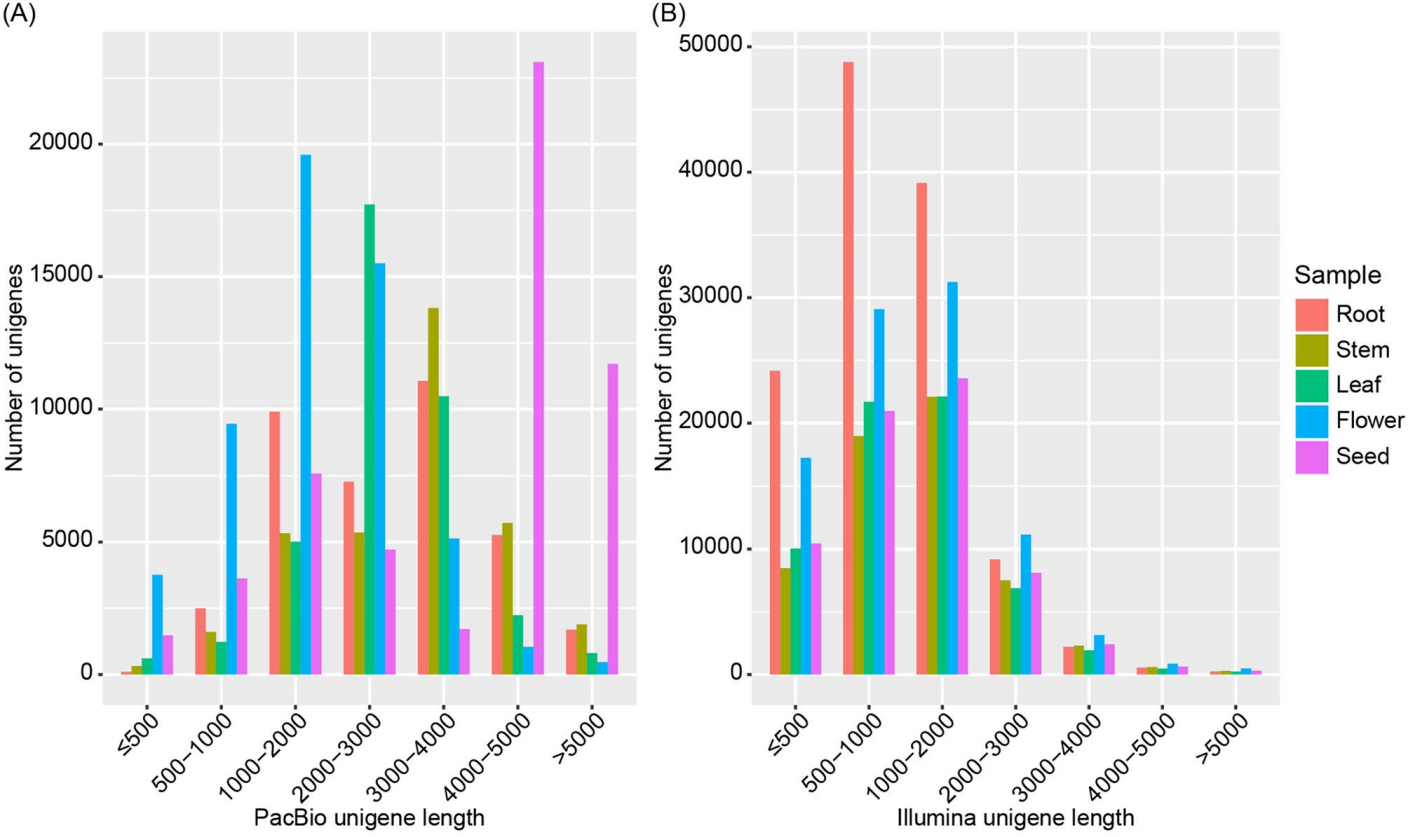
Length distribution of unigenes from PacBio SMRT sequencing (**A**) and Illumina RNA-Seq (**B**) across five organs.

Based on PacBio SMRT sequencing, 3,751,089, 3,434,452, 3,900,180, 8,535,019, and 4,435,846 subreads were generated for root, stem, leaf, flower, and seed, with a N50 of 3040, 3367, 2611, 2198, and 4584 bp, respectively (Table S1; Fig. S1). Subreads were processed to generate circular consensus sequences (CCSs). By detecting the primers and poly(A) tail, 238,196, 232,290, 211,535, 257,905, and 231,877 full-length non-chimeric (FLNC) reads were identified for root, stem, leaf, flower, and seed, with a mean length of 2633, 3070, 2561, 1746, and 3762 bp, respectively (Table S2; Fig. S2). After Iterative Clustering for Error Correction (ICE) clustering, polishing, base correction, de-redundancy, and non-plant sequences filtering, 37,789, 34,034, 38,100, 54,937, and 53,906 unigenes were retained for root, stem, leaf, flower, and seed, respectively, with an average unigene length of 1802–3786 bp and N50 of 2238–4707 bp (Table S2). The length of most unigenes from five organs exceeded 2000 bp, accounting for 68.88% of the total number (Table S3; Fig. 1A). Based on Benchmarking Universal Single-Copy Orthologs (BUSCO) assessment, about 88.1% (single-copy: 353; duplicated: 916) of the 1440 core embryophyte genes were found to be complete (90.6% were present when counting fragmented genes), suggesting the high integrity of the *M. micrantha* transcriptome (Fig. S3).

### De novo assembly of Illumina RNA-Seq data

Based on Illumina RNA-Seq, 43.23, 40.27, 41.01, 65.85, and 41.09 million clean reads were obtained for root, stem, leaf, flower, and seed, respectively, with Q20 exceeding 96.72%. Using Trinity software, clean reads were de novo assembled into 124,238, 60,232, 63,370, 93,229, and 66,411 unigenes for root, stem, leaf, flower, and seed. After filtering non-plant sequences, 124,233, 60,232, 63,370, 93,228, and 66,410 unigenes were finally retained for the five organs, respectively (Table S4). The length of most unigenes (84.70%) was shorter than 2000 bp (Table S3). In addition, the average length and N50 of unigenes generated by Illumina RNA-Seq were 1067–1312 bp and 1336–1685 bp, respectively, which were shorter than that from PacBio SMRT sequencing (Table S4; Fig. 1B).

### Functional annotation

To obtain a comprehensive functional annotation of *M. micrantha* transcriptome, unigenes generated by PacBio SMRT sequencing were annotated in seven public databases, including NCBI non-redundant nucleotide sequences (NT), NCBI non-redundant protein sequences (NR), Gene Ontology (GO), Eukaryotic Orthologous Groups (KOG), Kyoto Encyclopedia of Genes and Genomes (KEGG), Swiss-Prot, and Pfam protein families. For root, stem, leaf, flower, and seed, 35,714 (94.51%), 32,614 (95.83%), 36,134 (94.84%), 49,197 (89.55%), and 50,962 (94.54%) unigenes were annotated to at least one database, respectively, suggesting that our transcriptome is well annotated and that most of unigenes may be functional (Table 1).

**Table 1:** Statistics of annotation of full-length transcripts from five *M. micrantha* organs in seven databases.

Database	Root	Stem	Leaf	Flower	Seed
NR	33,847	30,793	35,085	48,112	50,152
SwissProt	31,050	28,857	31,673	40,966	43,513
GO	24,690	22,808	25,706	31,867	32,978
KOG	23,212	20,772	23,223	29,560	35,666
KEGG	14,305	13,539	15,140	21,384	20,326
NT	25,505	24,515	26,729	35,787	39,553
Pfam	24,690	22,808	25,706	31,867	32,978
At least one database	35,714	32,614	36,134	49,197	50,962
All databases	9268	8800	9406	12,693	13,001

Based on NR database annotation, the top three homologous species for the five organs were *Cynara cardunculus*, *Vitis vinifera*, and *Daucus carota* (Fig. S4). The top homologous species was a plant of the Asteraceae family. For the GO function annotation, “binding”, “catalytic activities”, “metabolic process”, “cellular process”, “cell”, and “cell part” were functional categories with the most abundant unigenes (Fig. S5). In addition, numerous unigenes were assigned to “response to stimulus”, “response to biotic stimulus”, and “response to oxidative stress” category (Table S5). Positive response to stress stimuli is an important strategy for invasive plants to adapt to the environment. In the KEGG annotation, the top two pathways with the most abundant unigenes were “carbohydrate metabolism” and “translation”. Furthermore, “energy metabolism” and “environmental adaptation” were also worthy of attention, which are important pathways responsible for energy supply and stress responses (Fig. S6).

### TFs identification and AS analysis

Using the iTAK pipeline, 1776 (root), 1293 (stem), 1627 (leaf), 2529 (flower), and 1733 (seed) unigenes were identified as TFs, which were classified into 68 families (Table S6). C3H (884), C2H2 (525), and bHLH (501) were the most abundant TF families (Fig. S7A). In addition, MYB (333) TFs were also found in the five organs. The differential expression levels of the top 15 TF families were further characterized. We found that the top 15 TF families had a certain amount of expression in the five organs of *M. micrantha* (Fig. S7B).

For root, stem, leaf, flower, and seed, 3300, 2324, 3219, 4730, and 3740 unique transcript models (UniTransModels) were constructed, among which the UniTransModels containing two isoforms were the most common (Fig. S8A). There were 329, 270, 358, 336, and 537 AS events identified in root, stem, leaf, flower, and seed, respectively. Retained introns (RIs) were detected as the most abundant AS event in all five organs, followed by alternative 3′ splice sites (A3) and alternative 5′ splice sites (A5). Mutually exclusive exons (MX) were the least frequent event (Fig. S8B).

### Gene expression analysis

**Figure 2:**
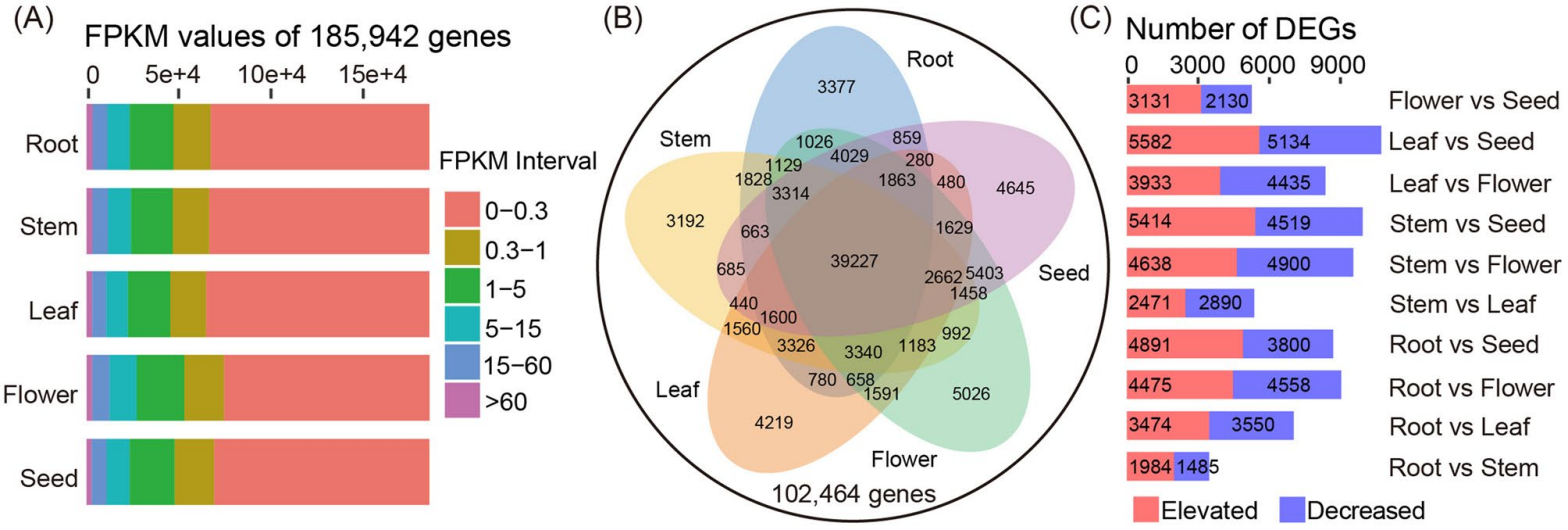
Gene expression patterns in five *M. micrantha* organs. (**A**) The FPKM interval distribution in the five organs. (**B**) Venn diagram of the number of unigenes expressed in five organs. (**C**) Number of differentially expressed genes in each pairwise comparison of five organs.

The number of unigenes in different expression level intervals was similar across the five organs (Fig. 2A). Using FPKM > 0.3 as the threshold for unigene expression, the total number of unigenes expressed in the five organs was 102,464 (Fig. 2B). Among them, 39,227 unigenes were co-expressed in all five organs. The information of differentially expressed genes (DEGs) identified in pairwise comparisons among the five organs is listed in Table S7. In total, 21,161 DEGs were identified among the five organs (Fig. S9). The number of DEGs between the five organs varied from 3469 (root vs stem) to 10,716 (leaf vs seed) (Fig. 2C). Notably, 933, 428, 1410, 1018, and 1292 DEGs showed significant higher expression in root, stem, leaf, flower, and seed, respectively (Figs. S10 and S11).

### KEGG enrichment of unigenes with higher expression in each organ

**Figure 3:**
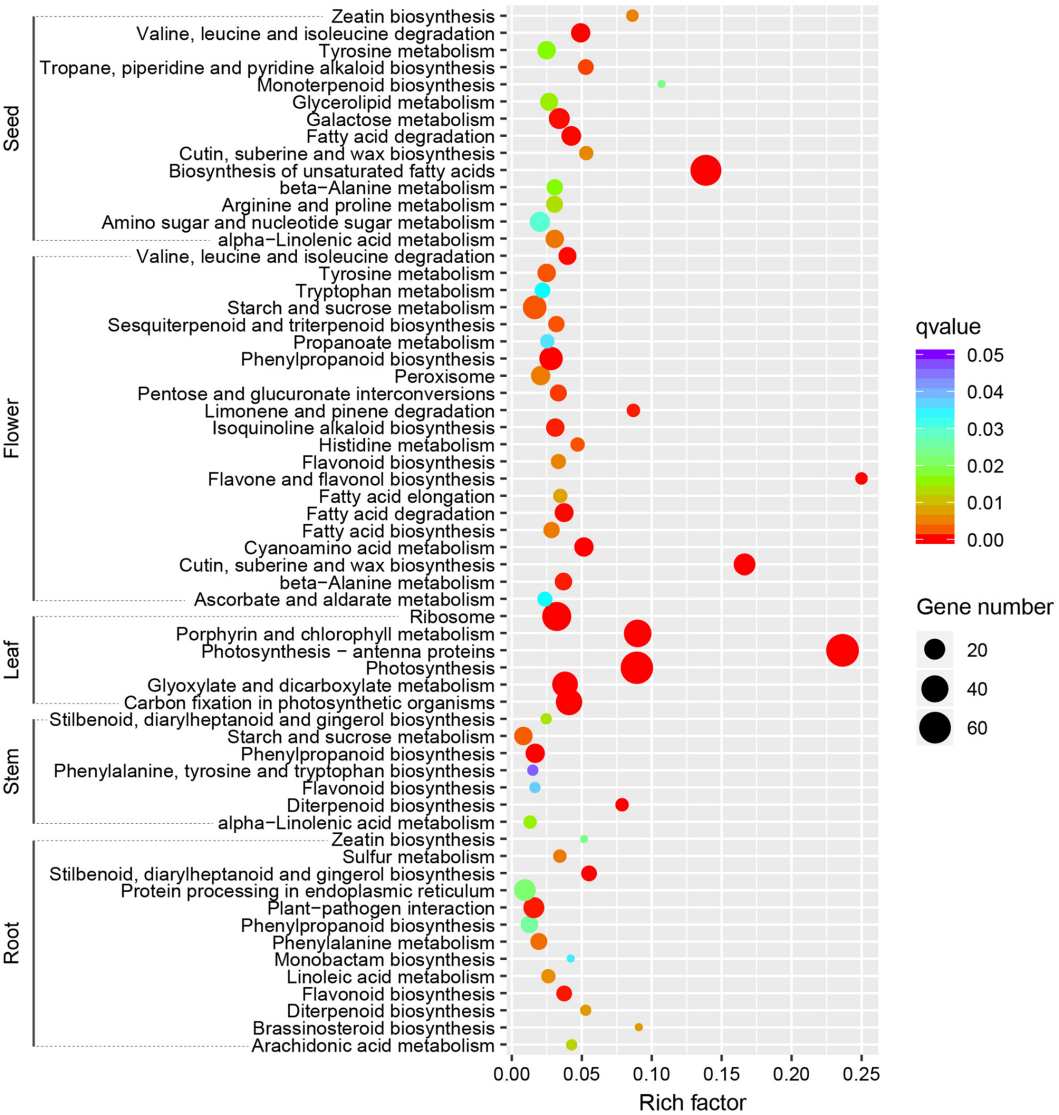
The Kyoto Encyclopedia of Genes and Genomes (KEGG) pathway enrichment analysis of unigenes with higher expression in each organ. The significantly enriched pathways with corrected *p*-value (*q* value) < 0.05 are shown. Number indicates the size of the dot, describing the number of unigenes enriched in the pathway. The color bar represents the *q* value and indicates significance of the enrichment. The blue represents higher value, and the red represents lower value.

According to the KEGG enrichment analysis results, there were obvious differences in enriched pathways in the five organs (Table S8; Fig. 3). The unigenes with higher expression in root were mainly enriched to defense response and protein processing pathways, such as “plant-pathogen interaction” and “protein processing in endoplasmic reticulum”. In stem, unigenes with higher expression were predominantly enriched to pathways related to the secondary metabolite, sugar, and terpenoid biosynthesis, such as “phenylpropanoid biosynthesis”, “starch and sucrose metabolism”, and “diterpenoid biosynthesis”. In flower, unigenes with higher expression were mainly related to “starch and sucrose metabolism”, “phenylpropanoid biosynthesis”, and “cutin, suberine, and wax biosynthesis”. The unigenes with higher expression in seed were mainly enriched in three fatty acid and sugar metabolism pathways, namely “biosynthesis of unsaturated fatty acids”, “galactose metabolism”, and “amino sugar and nucleotide sugar metabolism”. The unigenes with higher expression in leaf were significantly enriched in photosynthesis pathways, including “photosynthesis-antenna proteins”, “photosynthesis”, “porphyrin and chlorophyll metabolism”, and “carbon fixation in photosynthetic organisms”, which are important for the photosynthesis of *M. micrantha*.

### Discovery of gene families related to stress response and adaptation

**Figure 4:**
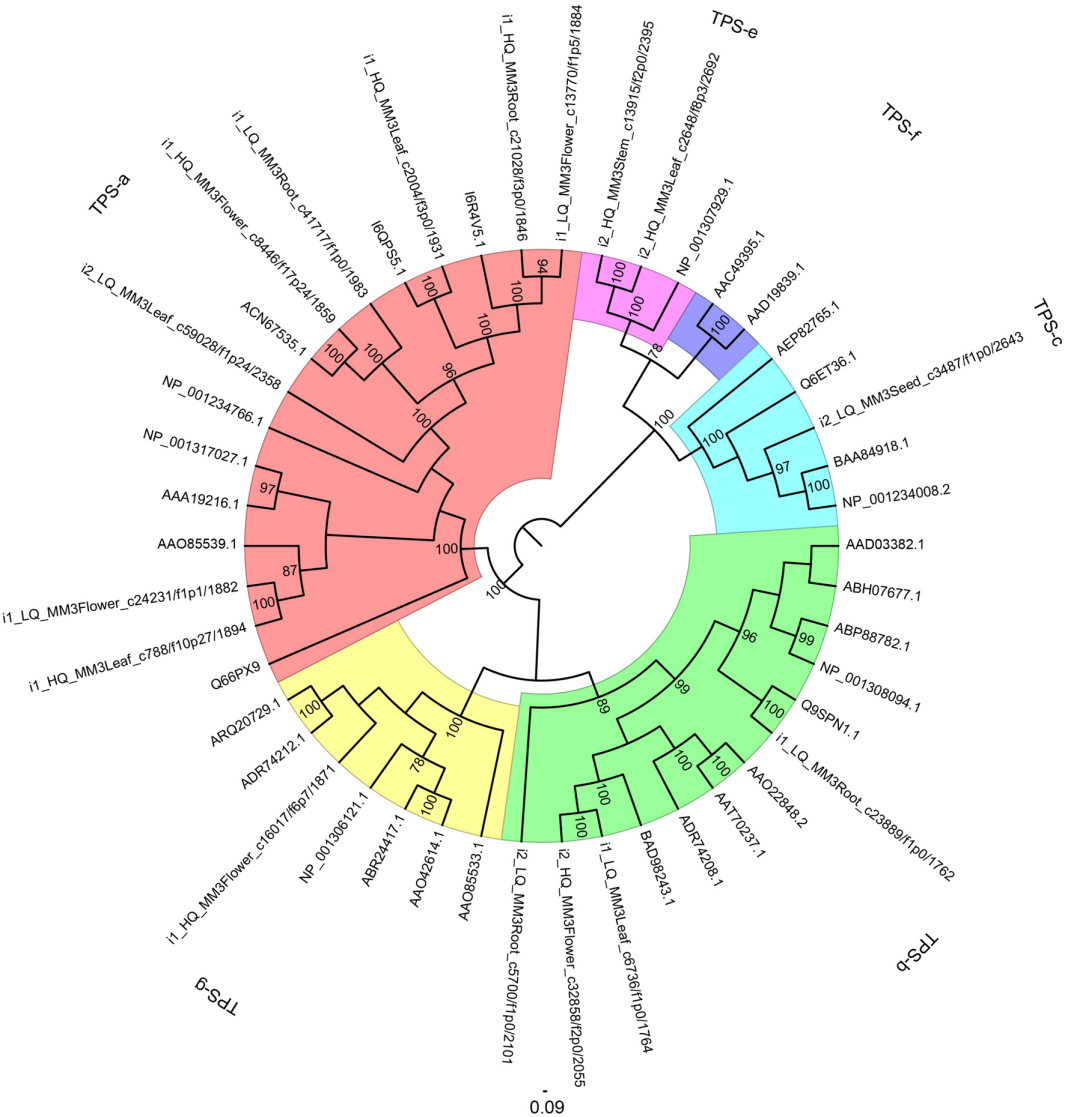
Phylogenetic analysis of terpene synthase (TPS) sequences from *M. micrantha* and other angiosperms.

For root, stem, leaf, flower, and seed, 62, 26, 59, 37, and 12 unigenes were identified as putative TPSs, respectively. Among them, most TPSs belonged to TPS-a subfamily (61 unigenes) and TPS-b subfamily (65), and only a small amount of TPSs were classified as TPS-e (7), TPS-f (1), and TPS-g subfamily (5) (Tables S9 and S10). The neighbor-joining (NJ) tree showed that TPS sequences were classified into six clades, among which TPS sequences from *M. micrantha* were clustered in TPS-a, TPS-b, TPS-c, TPS-e, and TPS-g clade (Fig. 4). Most sequences of TPS-a and TPS-b subfamily had higher expression in root, leaf, or flower of *M. micrantha*, and the rest TPS subfamilies were expressed most highly in different organs of *M. micrantha*.

**Figure 5:**
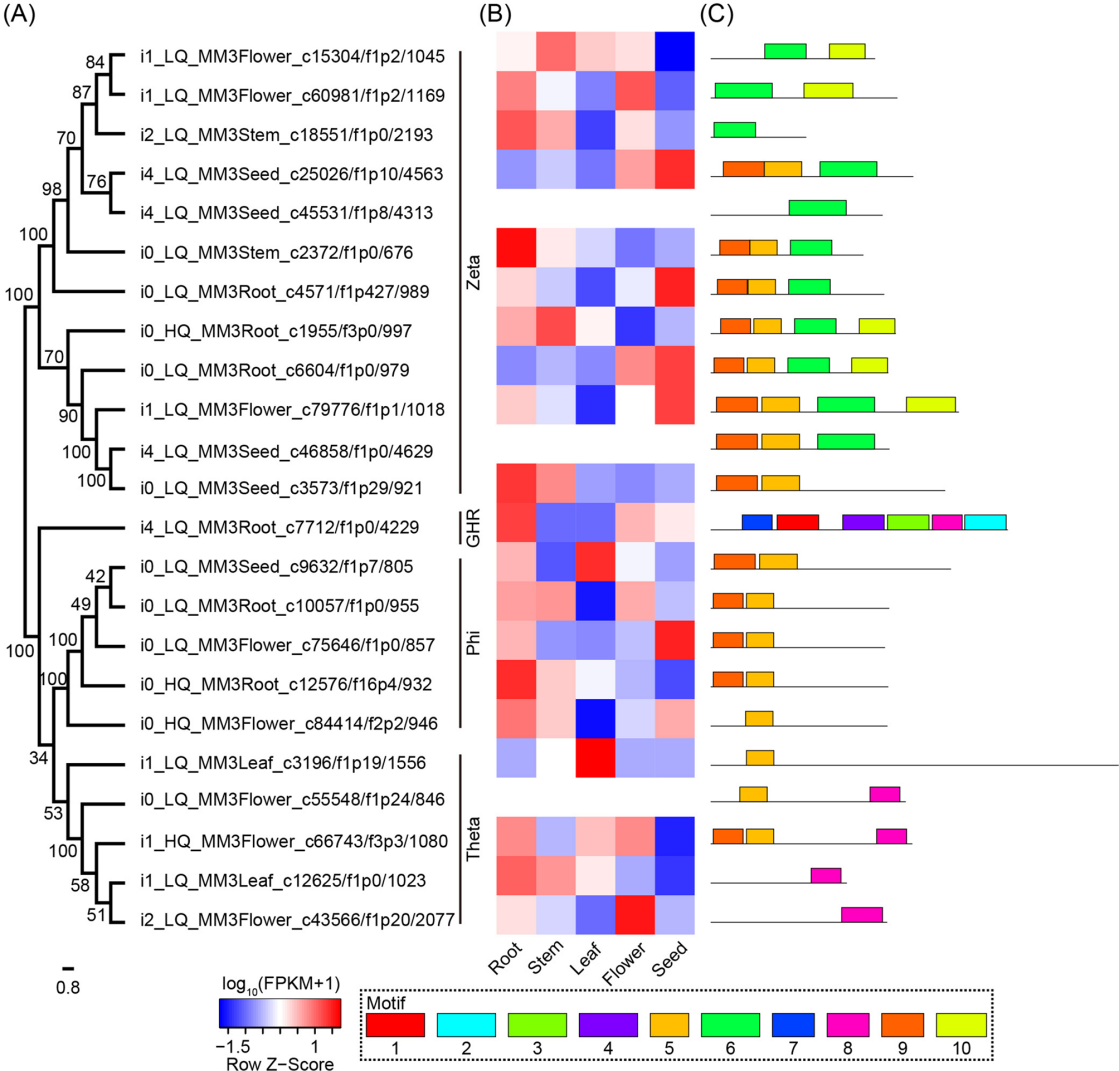
Neighbor-joining (NJ) phylogenetic tree, expression level, and conserved motif of glutathione S-transferase (GST). (**A**) NJ tree. (**B**) Expression heatmap. (**C**) Conserved motif.

A total of 172 unigenes annotated as GSTs were obtained from five organs, which were classified into Zeta (52 unigenes), Theta (37), Tau (18), Phi (19), glutathionyl-hydroquinone reductases (GHR, 15), dehydroascorbate reductase (DHAR, 11), Lambda (7), and Beta (1) subfamily and some unigenes with undetermined subfamily (Tables S9 and S11). The NJ tree showed that 23 GST sequences were divided into four groups, namely Zeta, GHR, Phi, and Theta subfamily (Fig. 5A). Ten conserved motifs were identified from 23 GST sequences, and motif distribution was obviously different among the four subfamilies (Fig. 5C). Sequences from GHR subfamily were mainly expressed in root, flower, and seed, while sequences distributed in Zeta, Phi, and Theta subfamilies had certain expression levels in the five organs of *M. micrantha* (Fig. 5B).

### Pathway related to environmental adaptation

**Figure 6:**
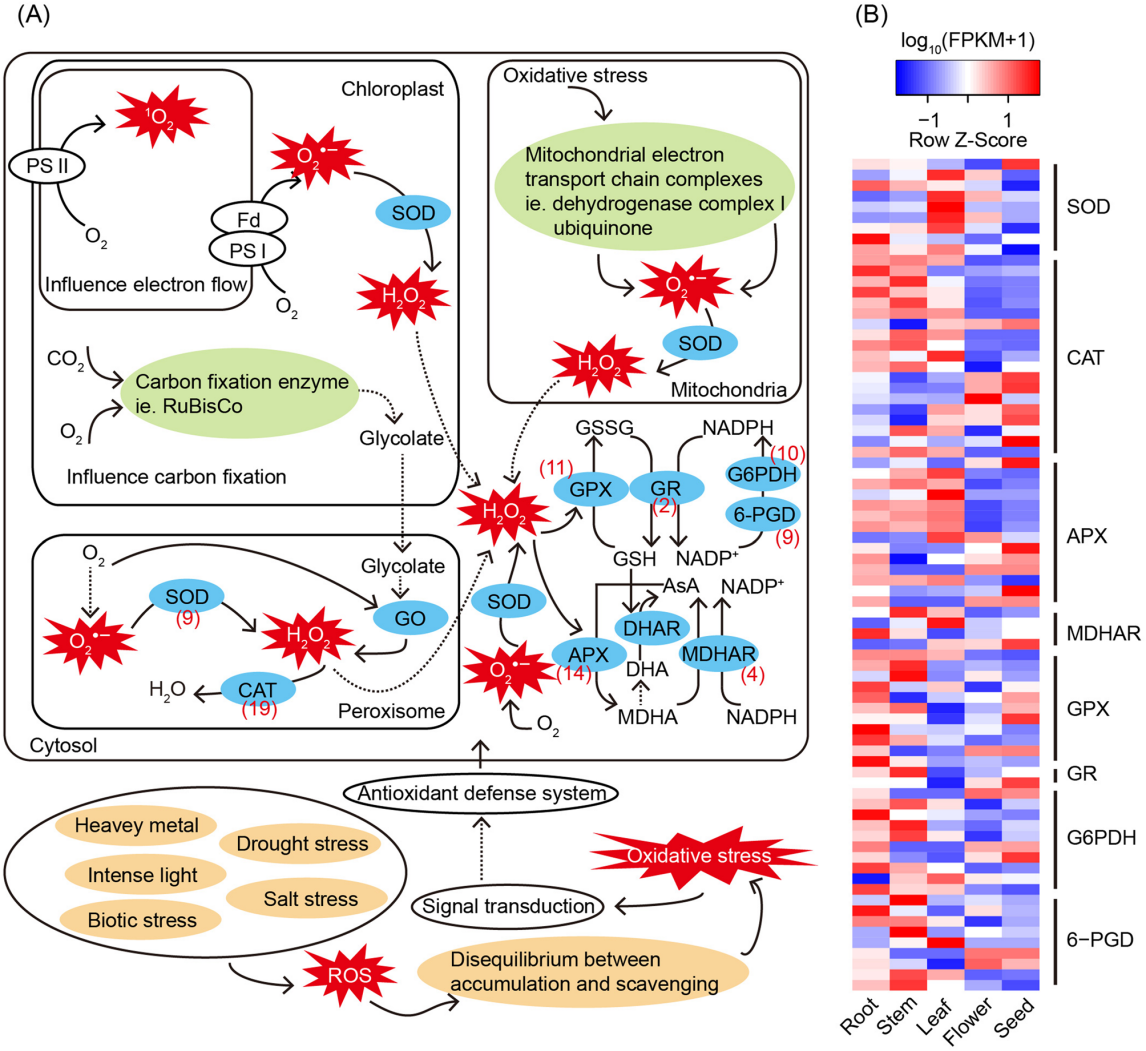
Antioxidant defense system in *M. micrantha* and the differentially expressed genes (DEGs) involved in the antioxidant defense in five organs of *M. micrantha*. (**A**) A simplified diagram of the antioxidant defense system. Numbers in brackets represent DEG numbers. (**B**) A heatmap of the DEGs involved in the antioxidant defense in five organs.

Timely scavenging of ROS under high photosynthetic efficiency and potential stresses is critical for the normal growth and development of *M. micrantha*. A total of 419 unigenes involved in antioxidant defense system were identified in the five organs, which included superoxide dismutase (SOD, 40 unigenes), catalase (CAT, 155), ascorbate peroxidase (APX, 67), monodehydroascorbate reductase (MDHAR, 19), glutathione peroxidase (GPX, 44), glutathione reductase (GR, 22), glucose-6-phosphate 1-dehydrogenase (G6PDH, 46), and 6-phosphogluconate dehydrogenase (6-PGD, 26) (Table S12). Among them, 78 unigenes were identified as DEGs in the five organs (Fig. 6A). SOD can directly oxidize superoxide radicals (O_2_^•−^) to hydrogen peroxide (H_2_O_2_), which is further converted into H_2_O through CAT enzyme. The DEGs responsible for the synthesis of SOD and CAT exhibited higher expression levels in root, stem, leaf, and seed than that in flower. Consistently, the other antioxidant enzymes used for scavenging of toxic H_2_O_2_, such as APX, MDHAR, GPX, GR, G6PDH, and 6-PGD, were also be found to be predominantly expressed in root, stem, leaf, and seed (Fig. 6B).

**Figure 7:**
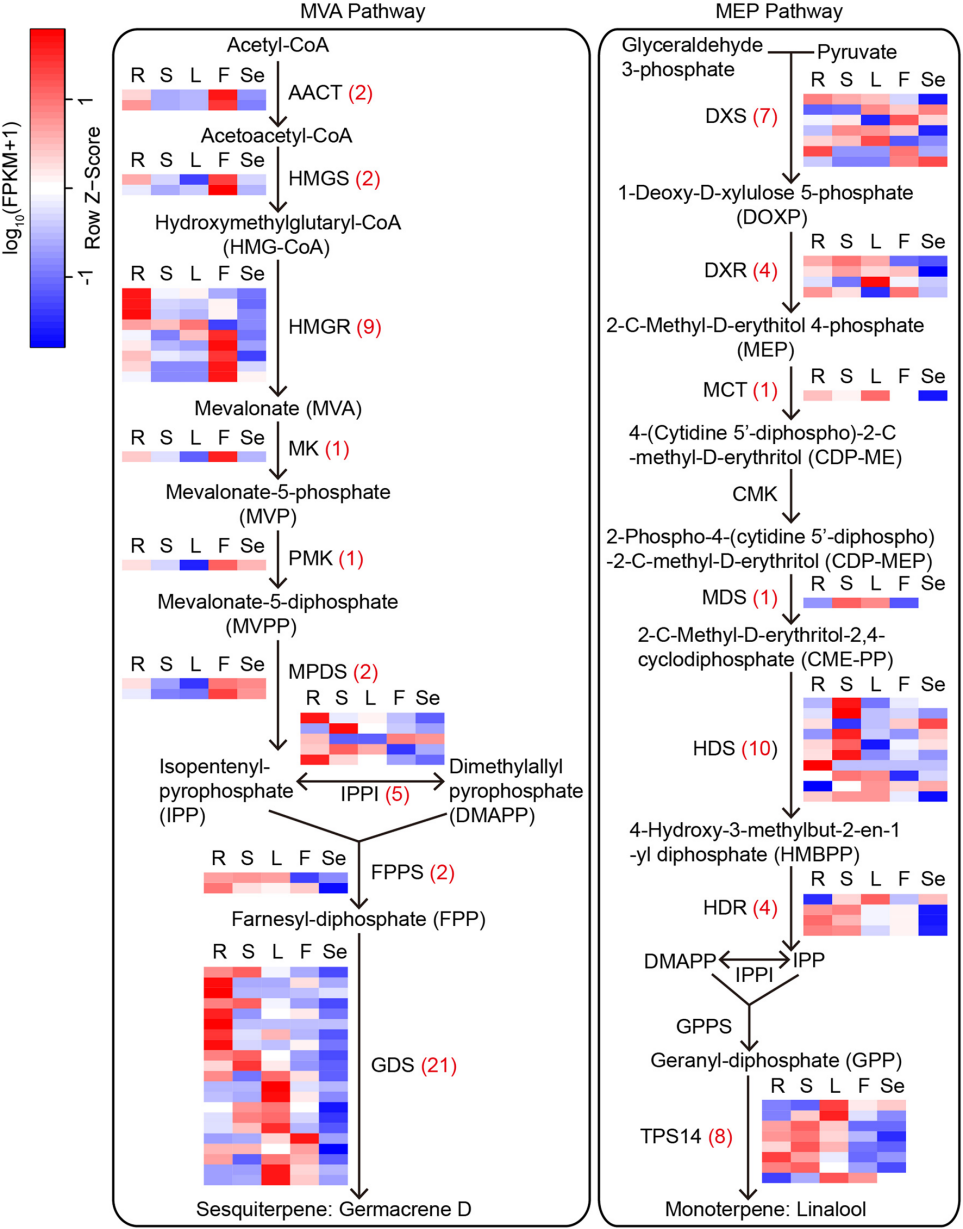
MVA and MEP pathways^22^ for terpenoid biosynthesis in *M. micrantha* and the differentially expressed genes (DEGs) involved in the terpenoid biosynthesis in five organs of *M. micrantha*. Numbers in brackets represent DEG numbers. The heatmap represents the expression level of DEGs in five organs. R: root; S: stem, L: leaf, F: flower, and Se: seed.

Plants produce a variety of terpenoids in response to biotic and abiotic stresses. A total of 385 unigenes from the five organs were assigned to MVA and MEP pathways^22^ (Table S13; Fig. 7). In the MVA pathway, acetyl-CoA acetyltransferase (AACT, 20 unigenes), 3-Hydroxy-3-methylglutaryl-CoA synthase (HMGS, 16), 3-Hydroxy-3-methylglutaryl-CoA reductase (HMGR, 49), mevalonate kinase (MK, 4), phosphomevalonate kinase (PMK, 3), diphosphomevalonate decarboxylase (MPDS, 6), isopentenyl-diphosphate delta-isomerase (IPPI, 13), and farnesyl diphosphate synthase (FPPS, 18) were identified as important components in the synthesis of sesquiterpene precursors. Germacrene D synthase (GDS) acts as a key terpene synthase, which can catalyze the precursors into sesquiterpene germacrene D. In this study, we identified 52 GDSs in five organs (Table S13). In the MEP pathway, 66, 6, 2, 12, 8, 41, 36, 13, 10, and 23 unigenes were annotated as 1-Deoxy-D-xylulose-5-phosphate synthase (DXS), 1-Deoxy-D-xylulose-5-phosphate reductoisomerase (DXR), 2-C-methyl-D-erythritol 4-phosphate cytidylyltransferase (MCT), 4-(cytidine 5-diphospho)-2-C-methyl-d-erythritol kinase (CMK), 2-C-methyl-D-erythritol 2,4-cyclodiphosphate synthase (MDS), 4-Hydroxy-3-methylbut-2-enyl-diphosphate synthase (HDS), 4-Hydroxy-3-methylbut-2-en-1-yl diphosphate reductase (HDR), IPPI, geranyl diphosphate synthase (GPPS), and (3S)-linalool synthase (TPS14), which were responsible for the biosynthesis of monoterpene linalool. Among the 385 unigenes identified in the MVA and MVP pathways, 80 unigenes were determined as DEGs in the five organs. Nearly half of the DEGs (38 DEGs, accounting for 47.50%) displayed lower expression levels in the seed than that in the other four organs, while the number of DEGs expressed most highly was similar in the root (18 DEGs), stem (22), leaf (19), and flower (18) (Fig. 7).

## Discussion

In this study, we obtained a comprehensive overview of the transcriptomic profiles of the five *M. micrantha* organs based on the Illumina RNA-Seq and PacBio SMRT data. Previous transcriptome studies of *M. micrantha* used single or mixed vegetative organ samples and second-generation technology^16–18^. The average length of unigenes produced by these studies (585–1077 bp) were shorter than the 1802–3786 bp from the PacBio transcriptomic data of the five organs. This result indicates that PacBio SMRT sequencing has a better capability to capture long transcripts. In particular, PacBio SMRT sequencing captured more transcripts longer than 4000 bp in the seed transcriptome of *M. micrantha*, which may be partly related to the higher proportion of long transcripts in subread and FLNC read dataset. Moreover, our analysis contained vegetative organs and reproductive organs, which makes up for the lack of transcriptome data of reproductive organs and increases the transcript diversity of *M. micrantha*. The independent library construction of different organs provides putative opportunity for the discovery of organ-specific transcripts and gene expression profiles, and is conducive to exploring the potential role of different organs in the environmental adaptation of invasive plants. In addition, 89.55–95.83% of the PacBio unigenes from five *M. micrantha* organs had homology with sequences in seven public databases. The annotation rates were higher than that reported in previous next-generation transcriptome studies^16–18^, indicating that PacBio unigenes could promote annotation and provide more information for *M. micrantha* transcriptomes.

TFs are important regulatory proteins that control the gene expression, which play key roles in the regulation of physiological functions and the response of plants to various stresses. In this study, we found 1293–2529 unigenes encoding TFs in the five organs, which was comparable to those of model plant *Arabidopsis thaliana*^23^. C3H, C2H2, and bHLH were the most abundant TF families in the five organs. Consistently, these three types of TF families have also been found to be abundant in the transcriptome of other plants, such as invasive *Phytolacca americana* and non-invasive bermudagrass (*Cynodon dactylon*)^24,25^. Gene duplication event plays a significant role in the amplification of C3H, C2H2, and bHLH gene families^26–28^. The phenomenon of C3H, C2H2, and bHLH families being the most abundant may be the result of a higher number of gene duplication events in these gene families during the evolution of the *M. micrantha* genome. Furthermore, the existence of a large number of C3H, C2H2, and bHLH TFs may be related to their potential functions. C3H and C2H2 belong to zinc finger proteins, which can regulate the expression of stress-related genes and increase the resistance to salinity, drought, excessive light, and oxidative stress^29,30^. bHLH TFs are one of the most abundant regulatory protein families, involving in plant development, monoterpene linalool biosynthesis, and stress response^31,32^. In addition, MYB TFs were also found in the five organs. MYB TFs have been shown to involve in sesquiterpene germacrene D production^33^. The expression analysis showed that C3H, C2H2, bHLH, and MYB families were expressed in all five organs, suggesting that they may play important roles in organ development and defense response in *M. micrantha*. Our data provide a rich resource for further study on the potential regulatory roles of TFs in *M. micrantha*.

Increasing numbers of studies have shown that AS may occur in some genes under adverse environmental stress. For example, about 49% of intron-containing genes in *Arabidopsis* experienced AS events under salt stress^34^. The activation of AS promoted the adaptation of *Spartina alterniflora* to the high-salt environment^21^. The information of AS in *M. micrantha* is still unclear. In our study, we identified 1830 AS events in the five organs. RIs were identified as the most abundant AS events, which was in line with the patterns of AS reported in plants^35^. During the process of invasion, AS may participate in the adaptation of *M. micrantha* to invasive environment. In this sense, the roles of AS in the regulation of *M. micrantha* adaptation should be further studied.

Unigenes with higher expression in each organ were significantly enriched into distinct functional pathways. Unigenes with higher expression in root were enriched into “plant-pathogen interaction” pathway. Several studies have shown that “plant-pathogen interaction” participates in the response to pathogens attack in plant^36^. Unigenes with higher expression in stem were predominantly assigned to “diterpenoid biosynthesis” and “starch and sucrose metabolism” pathway. “Diterpenoid biosynthesis” is linked to gibberellins biosynthesis, which has a great effect on stem elongation^37^. Starch and sucrose are the main energy supplier in higher plants, which are implicated in the metabolism and growth of plants. The decrease of starch and sucrose content shortens the growth cycle and reduces vegetative growth in *Lactuca sativa*^38^. Photosynthesis-related pathways, such as “carbon fixation in photosynthetic organisms” and “photosynthesis”, were significantly enriched in leaf. These functional categories have significant implications for the photosynthesis of *M. micrantha*. Unigenes with higher expression in flower were enriched in “phenylpropanoid biosynthesis” and “cutin, suberine and wax biosynthesis” pathways. Phenylpropanoids are precursors of flavonoids that play vital roles in flower development and the defense against oxidative stress and abiotic stimuli^39^. “Cutin, suberine and wax biosynthesis” may be related to flower organ development in transgenic *Arabidopsis* plants^40^. Unigenes with higher expression in seed were mainly enriched in the “biosynthesis of unsaturated fatty acids” pathway. Unsaturated fatty acids play crucial roles in multiple biological processes, enabling plant to respond to biotic and abiotic stresses^41^. Taken all together, these results suggest that certain functional pathways may have crucial implications regarding the environmental adaptation and organ development of *M. micrantha*.

Plants possess a variety of TPSs, which are responsible for the biosynthesis of terpenoids that function in growth, development, and stress responses. In this study, 196 TPS unigenes were identified from *M. micrantha* transcriptome, which was comparable with those reported in some angiosperms^42^. The phylogenetic tree showed that *M. micrantha* TPS sequences were divided into TPS-a, TPS-b, TPS-c, TPS-f, and TPS-g subfamilies, which was congruent with the result of phylogenetic analysis of TPS in angiosperms^43^. Most TPSs in angiosperms are from the TPS-a and TPS-b subfamilies, while fewer TPSs belong to TPS-c, TPS-e/f, and TPS-g subfamilies^44^. TPS-c and TPS-e/f subfamilies contain sesqui-, mono-, and other diterpene synthases^43^. TPS-a, TPS-b, and TPS-g are angiosperm-specific subfamilies, comprising genes of specialized sesqui-, mono-, and diterpene biosynthesis, which participate in response to insect damage and abiotic stress^42,45^. *Mikania micrantha* TPS-a and TPS-b subfamilies were mainly highly expressed in the root, leaf, or flower, suggesting their significant implications for root, leaf, and flower. The function of the TPS gene family has been characterized in invasive plants. For example, *EaTPS1* is likely involved in the biosynthesis of cadinene sesquiterpenes, which show potent antifeedant activity against insect *Spodoptera exigua*, indicating that TPS genes have important defensive roles in *E. adenophorum*^46^.

GSTs are one of the largest gene families and have functions in protecting plants from abiotic and biotic stress. GSTs are classified into distinct subfamilies, among which Zeta and Theta subfamilies exist in both plants and animals, whereas Tau and Phi subfamilies are specific to plants^47^. The overexpression of Tau and Phi GSTs contributes to plant tolerance to a variety of abiotic stresses, such as dehydration, high salinity, heavy metals, and oxygen stress^48,49^. Zeta GSTs enhance plant growth and germination at low temperature, and Theta GSTs confer tolerance to abiotic and oxidative stresses in tobacco plants^50,51^. The GHR and DHAR subfamilies are also important components of the GST gene family, whose members are up-regulated or differentially regulated under high-temperature, drought, and other abiotic stresses^52,53^. In our study, a total of 172 unigenes annotated as GSTs were identified in the five organs of *M. micrantha*, mainly including members of the Zeta, Theta, Tau, Phi, GHR, and DHAR subfamilies. Moreover, the members of Zeta, Theta, Phi, and GHR subfamilies were found to highly express in the five organs. Given the extensive functions of these GST subfamilies, it can be inferred that they may play an important role in *M. micrantha*.

Alien plants are expected to be exposed to multiple biotic and abiotic stresses when adapting to invasive environments. Stresses are often accompanied by an increase in production of ROS, leading to an imbalance of intracellular redox homeostasis and disrupting normal metabolism^13^. Plants have evolved antioxidant defense system to protect them from ROS damage. The protective roles of antioxidant enzymes in enhancing plant tolerance to various adverse environments have been widely proved. The SOD and CAT activities of *Ailanthus altissima* were significantly inducted under drought and salt stress^54^. Stress stimulus may induce the increase of glutathione and ascorbate content, and these two non-enzymatic antioxidants are important components for responding to different abiotic stresses^13^. Antioxidant enzyme APX, MDHAR, GPX, GR, G6PDH, and 6-PGD have been involved in the regeneration of glutathione and ascorbate, enhancing the ROS scavenging capacity and stress adaptability of plants^55,56^. Here, we reported unigenes encoding antioxidant enzyme SOD, CAT, APX, MDHAR, GPX, GR, G6PDH, and 6-PGD, indicating that antioxidant defense system is applied in *M. micrantha*, and that these enzymes may be conducive to improve the tolerance to drought stress, high light, and biotic infestation of *M. micrantha*^57,58^. Most DEGs in the antioxidant defense system exhibited higher expression levels in root, stem, leaf, and seed than in flower, suggesting that root, stem, leaf, and seed have strong ability to respond to adverse stresses.

*Mikania micrantha* plants produce rich terpenoids, especially monoterpenes and sesquiterpenes, which play an important role in plant growth and development and stress response^59^. In our transcriptome data, we found unigene sequences of key genes in the MVA and MEP pathways. Eighty unigenes were differentially expressed in five organs, and the majority of them (77 unigenes) were predominantly expressed in root, stem, leaf, and flower, indicating that root, stem, leaf, and flower form the main organs of terpenoid synthesis and accumulation in *M. micrantha*^5^. In addition, the enzymes in these two synthetic pathways and their roles in growth, development, and stress response in plants have been well established. Gene encoding DXS was demonstrated to respond to wounding stress by increasing terpene biosynthesis in invasive weed *S. elaeagnifolium*^11^. Genes encoding AACT, HMGS, HMGR, MPDS, IPPI, FPPS, DXS, DXR, MCT, CMK, MDS, HDS, HDR, and GPPS were found to involve in plant development and growth, pathogen resistance, and abiotic stress tolerance^59^. Germacrene D and linalool are important constituents of sesquiterpenes and monoterpenes in plants. Germacrene D possesses significant allelopathic activity, which can inhibit the seed germination and the growth of other plants^60^. Meanwhile, germacrene D is an important response factor to insect attack and salt stress^61,62^. Linalool shows significant phytotoxic and antimicrobial activities^63^. In our study, we identified terpene synthases for the synthesis of germacrene D and linalool, which is of great significance for protecting *M. micrantha* from herbivore damage and insect infestation and enhancing its stress adaptability.

In summary, we reported the full-length transcriptomes of five *M. micrantha* organs by applying the PacBio SMRT and Illumina RNA-Seq technologies. A total of 218,766 unigenes were obtained from five organs and were functionally annotated in seven public databases. Furthermore, TFs and AS events were identified. Gene expression patterns among the five organs and genes with higher expression in each organ were also analyzed. KEGG enrichment analysis of unigenes with higher expression indicated their special roles in environmental stress response and adversity adaptation in the various five organs. In addition, we identified gene families and pathways related to biotic and abiotic stress factors, including TPS and GST families, terpenoid biosynthesis pathway and antioxidant defense system. This study not only provides a large number of transcriptome resources for understanding the adaptation to invasive environment but will also contribute to further research on the adaptive evolution and functional genomics and offer a cue for promoting better prevention and control of this species.

## Materials and methods

### Plant materials and RNA extraction

The sampling work was approved by Sun Yat-sen University, and our research complies with the laws of the People’s Republic of China. The voucher specimen was identified by Xiaoxian Ruan and stored at the Herbarium of Sun Yat-sen University (voucher number: Ruan201705).

The *M. micrantha* plant was selected from the Neilingding Island, Shenzhen, Guangdong Province, China (22°24′35.13"N, 113°48′37.09"E). Its five organs (root, stem, leaf, flower, and seed) were collected from the same plant and stored in RNAfixer (Bioteke Corporation). Total RNA was extracted using the RNeasy Plant Mini Kit (Qiagen, Valencia, CA, USA) following the manufacturer’s protocol. RNA integrity was assessed using standard 1% agarose gel electrophoresis and an Agilent 2100 Bioanalyzer (Agilent Technologies, Santa Clara, CA, USA) and its concentration was determined using a Nanophotometer (Implen, Munich, Germany). High-quality RNA was used for cDNA synthesis and library construction.

### Illumina library preparation, sequencing, and de novo assembly

Illumina-based cDNA library for each organ was constructed using NEBNext Ultra RNA Library Prep Kit (NEB, USA), according to the manufacturer’s recommendations. The libraries were sequenced on an Illumina NovaSeq platform (Illumina, San Diego, CA, USA), generating 150 bp paired-end reads. The raw reads were quality-filtered using in-house Perl scripts to eliminate reads that contain adapters, more than 10% ambiguous bases (N), or more than 50% low-quality bases (Qphered ≤ 20). Clean reads were de novo assembled into unigenes using Trinity v2.4.0^64^. To improve the sequence quality, the unigene sequences were assessed by using BLAST searching against the NR database with an E-value cutoff of 1 × 10^–5^. The unigenes with top hits to non-plant organisms were excluded.

### PacBio library construction and SMRT sequencing

Total RNA for each of the five organs was used to construct the library separately following the PacBio SMRT sequencing experimental protocol. For each organ, three fractionated libraries (1–2 kb, 2–3 kb, and 3–6 kb) were constructed to eliminate loading bias. The libraries were sequenced on the PacBio Sequel platform (Pacific Biosciences, Menlo Park, CA, USA). Subreads were generated from the PacBio raw data by using SMRTlink v5.1 software (http://www.pacb.com/products-and-services/analytical-sofware/smrt-analysis/). The CCSs were obtained from subreads, and then classified into FLNC and non-full-length (nFL) reads based on the presence of 5′-primer, 3′-primer, and poly(A) tail. FLNC reads were clustered into consensus sequences by using ICE algorithm^65^. The consensus sequences were polished by nFL reads using the Arrow algorithm to obtain polished consensus sequences. The polished consensus sequences were further corrected by Illumina clean reads using LoRDEC software^66^, and redundant sequences were removed by using CD-HIT software (c = 0.95)^67^. Finally, the non-redundant transcripts (referred as unigenes) with top hits to non-plant organisms were excluded based on the results of NR annotation. The retained unigenes from five organs were merged and de-redundant to generate the reference transcriptome sequences of *M. micrantha*. The integrity of the reference transcriptome sequences was evaluated by BUSCO analysis with the core conserved gene set (embryophyta_odb9)^68^.

### Functional annotation

The unigenes of the five organs were functionally annotated in seven public databases. NT database annotation was conducted using BLAST 2.7.1 + with an E-value cutoff of 1 × 10^−5^. Searches against Pfam were performed by using the Hmmscan function of HMMER^69^. GO annotation was performed based on Blast2GO (http://www.blast2go.com)^70^ and in-house scripts. The NR, KOG, and Swiss-Prot database annotations were conducted using Diamond v0.8.36 software^71^ with an E-value cutoff of 1 × 10^−5^. KEGG pathway analysis was conducted using the KAAS (KEGG Automatic Annotation Server)^72^ and KEGG database^22^ with an E-value cutoff of 1 × 10^−10^.

### AS analysis and TFs identification

Coding GENome reconstruction Tool (Cogent v3.1, https://github.com/Magdoll/Cogent) was used to process non-redundant PacBio transcripts to create the k-mer profile. Based on the k-mer similarity, the De Bruijn graph method was used to reconstruct the transcripts into UniTransModels. GMAP-2017-06-20^73^ was applied to map transcripts to UniTransModels. Transcripts mapped to the same UniTransModels were examined to detect splicing junctions, and transcripts with the same splicing junctions were collapsed. Collapsed transcripts with different splicing junctions were defined as transcription isoforms. AS events were identified using SUPPA^74^ with default parameters. In addition, iTAK software^75^ was used to identify plant TFs in the five organs of *M. micrantha*.

### Gene expression quantification, DEGs identification, and enrichment analysis

The Illumina clean reads for each organ were mapped to the reference transcriptome sequences using Bowtie2^76^. The raw readcount value of each unigene in each organ was counted using RSEM software^76^, and then normalized to the FPKM (Fragments Per Kilobase of transcript per Million mapped reads). FPKM value was used to quantify the expression level of each unigene in each organ. Differentially expressed genes (DEGs) among the five organs were identified using the DEGSeq R package^77^. Benjamini–Hochberg multiple-testing correction was applied to adjust *p*-value for reducing the false discovery rate. Unigenes were assigned as DEGs with the criteria of corrected *p*-value < 0.005 and |log_2_(fold change)| > 1. KEGG pathways related to unigenes with higher expression in each organ were enriched using KOBAS v2.0^78^. Corrected *p*-values (*q* values) were used to determine the significance of KEGG enrichment, with corrected *p*-values < 0.05 considered significant.

### Analysis of gene families related to stress response and adaptation

Based on the functional annotations from NR, Swiss-Prot, KOG, and Pfam databases, the environmental stress-related gene families (TPSs and GSTs) were identified in *M. micrantha*. Furthermore, TPS and GST sequences were used to construct NJ phylogenetic trees using MEGA v5.10^79^. First, the TPS protein-coding sequences with complete domains and more than 500 amino acids were screened from the *M. micrantha* transcriptome. Second, sequences with more than 95% similarity were removed using CD-HIT^67^. Third, 30 TPS protein sequences of different angiosperms derived from GenBank were obtained (Table S14), which include all TPS subfamily in angiosperms. Finally, 16 TPS protein-coding sequences from *M. micrantha* transcriptome and 30 TPS protein sequences from GenBank were used to construct the TPS phylogenetic tree. The filtering of GST protein-coding sequences was consistent with that of TPS except for two conditions. The GST protein-coding sequences were more than 100 amino acids and had potential conserved motifs predicted by MEME Suite. MEME analysis parameters were set to -nmotifs 10, -minw 10, and -maxw 50. We finally used 23 GST protein-coding sequences from *M. micrantha* transcriptome to construct a GST phylogenetic tree. The family of GST protein-coding sequences was determined based on the classification in UniProt database (https://www.uniprot.org/). Using MAFFT v7^80^ with the default parameters, the protein sequences of these two gene families were separately aligned. 1000 bootstrap replications were performed to ensure the reliability of phylogenetic trees. Phylogenetic trees were visualized using FigTree v1.4.2^81^.

### Ethics statements

This study has not involved humans and animals.

## Supplementary Information

Supplementary Information.

Supplementary Table S6.

Supplementary Table S7.

Supplementary Table S8.

Supplementary Table S10.

Supplementary Table S11.

Supplementary Table S12.

Supplementary Table S13.

Supplementary Table S14.

## Data Availability

The Illumina raw data have been deposited in NCBI Sequence Read Archive (SRA) database as follows: root: SRR10596657; stem: SRR10596656; leaf: SRR10596655; flower: SRR10596654; and seed: SRR10596653. The PacBio transcriptome data were available under the Bioproject PRJNA592884 on the NCBI SRA database.
