# Supplementary material for: Full-length transcriptome analysis of multiple organs and identification of adaptive genes and pathways in Mikania micrantha
Source: Sci Rep. 2022 Feb 28;12:3272. doi: 10.1038/s41598-022-07198-0 (PMC8885683; doi:10.1038/s41598-022-07198-0)
Supplement: Supplementary file 1 — Supplementary Information. [file 41598_2022_7198_MOESM1_ESM.pdf]

Full-length transcriptome analysis of multiple organs and identification of adaptive genes and pathways in *Mikania micrantha*

Xiaoxian Ruan [1], Zhen Wang [1], Yingjuan Su [1,2,*] and Ting Wang [2,3,*]
[1] School of Life Sciences, Sun Yat-sen University, Guangzhou 510275, China;
[2] Research Institute of Sun Yat-sen University in Shenzhen, Shenzhen 518057, China;
[3] College of Life Sciences, South China Agricultural University, Guangzhou 510641, China;
[*] Correspondence: suyj@mail.sysu.edu.cn (Y.S.); tingwang@scau.edu.cn (T.W.).

Supplementary Figures

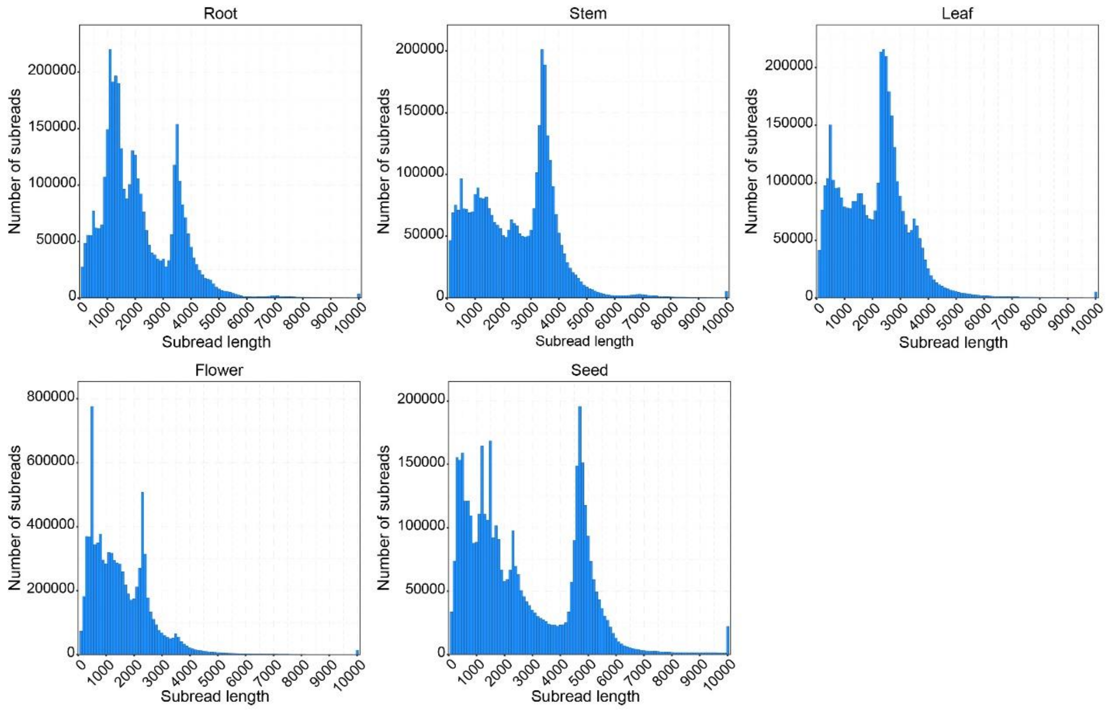

Figure S1. Length distribution of subreads in five *M. micrantha* organs.

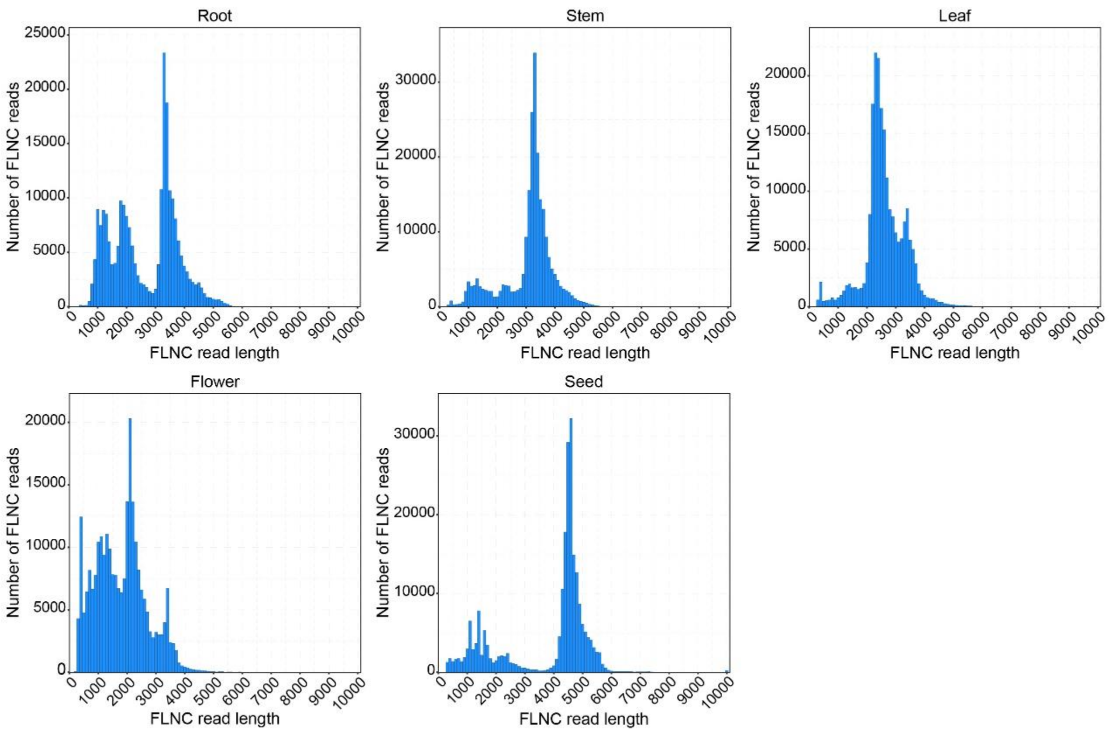

Figure S2. Length distribution of full-length non-chimeric (FLNC) reads in five *M. micrantha* organs.

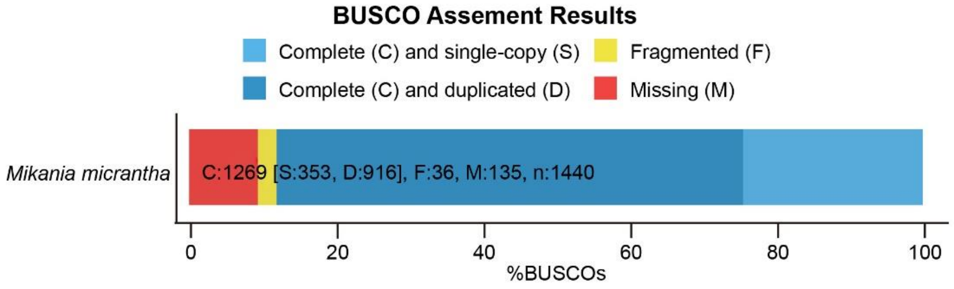

Figure S3. The result of the transcriptome completeness assessment based on Benchmarking Universal Single-Copy Orthologs (BUSCO) using the core conserved gene set (embryophyta_odb9).

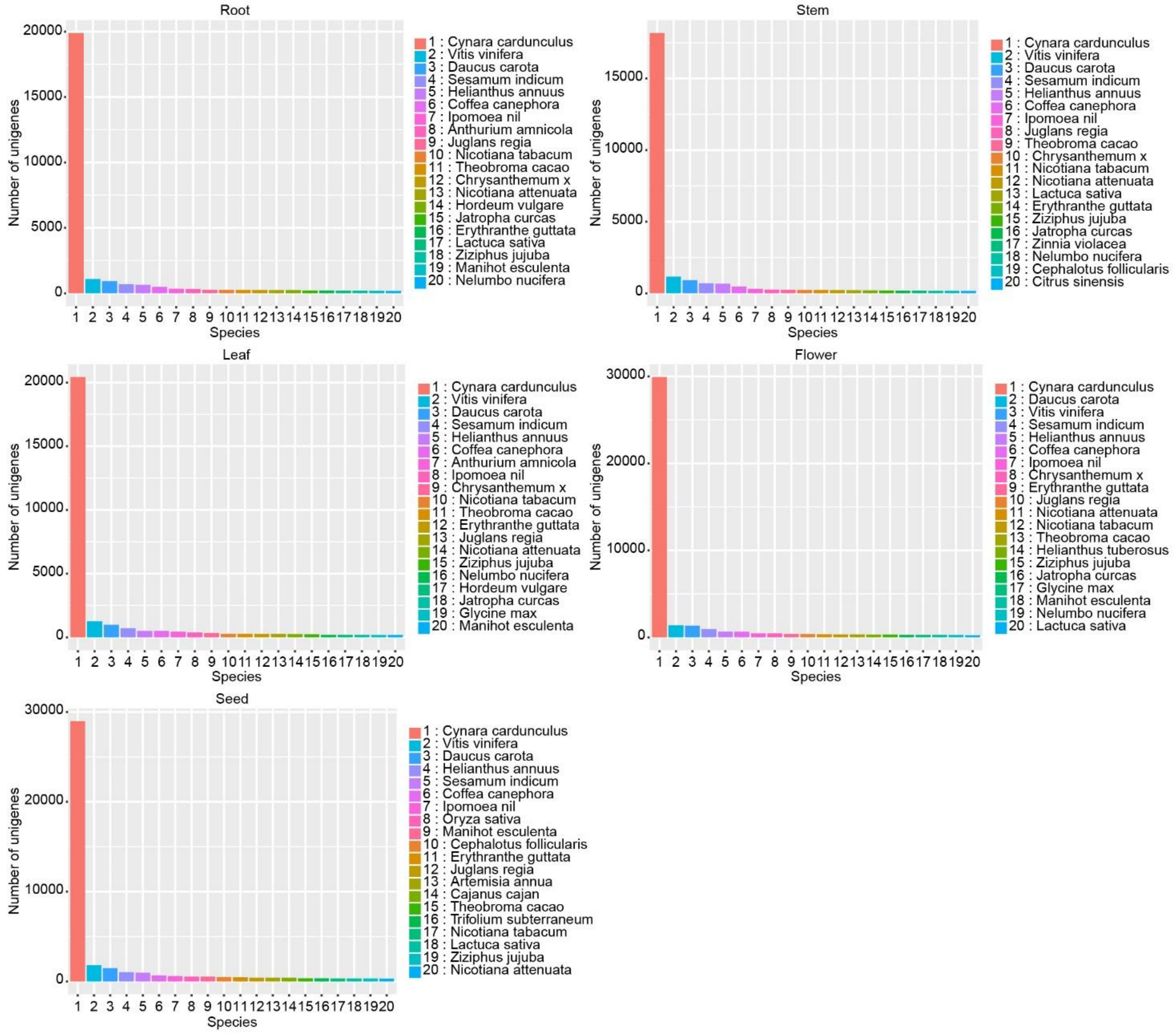

Figure S4. The distribution of the top twenty homologous species of unigenes in five *M. micrantha* organs.

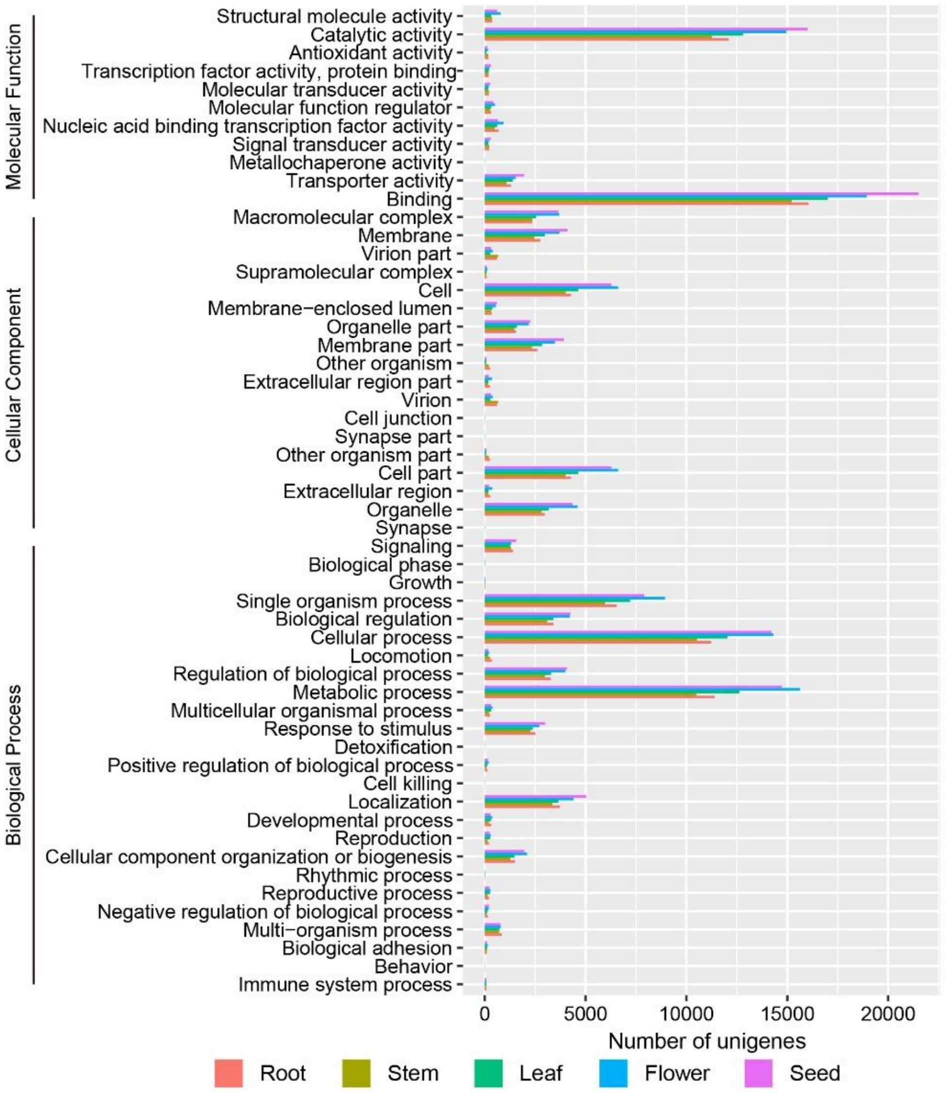

Figure S5. Function annotation and classification of Gene Ontology (GO) of unigenes for five *M. micrantha* organs.

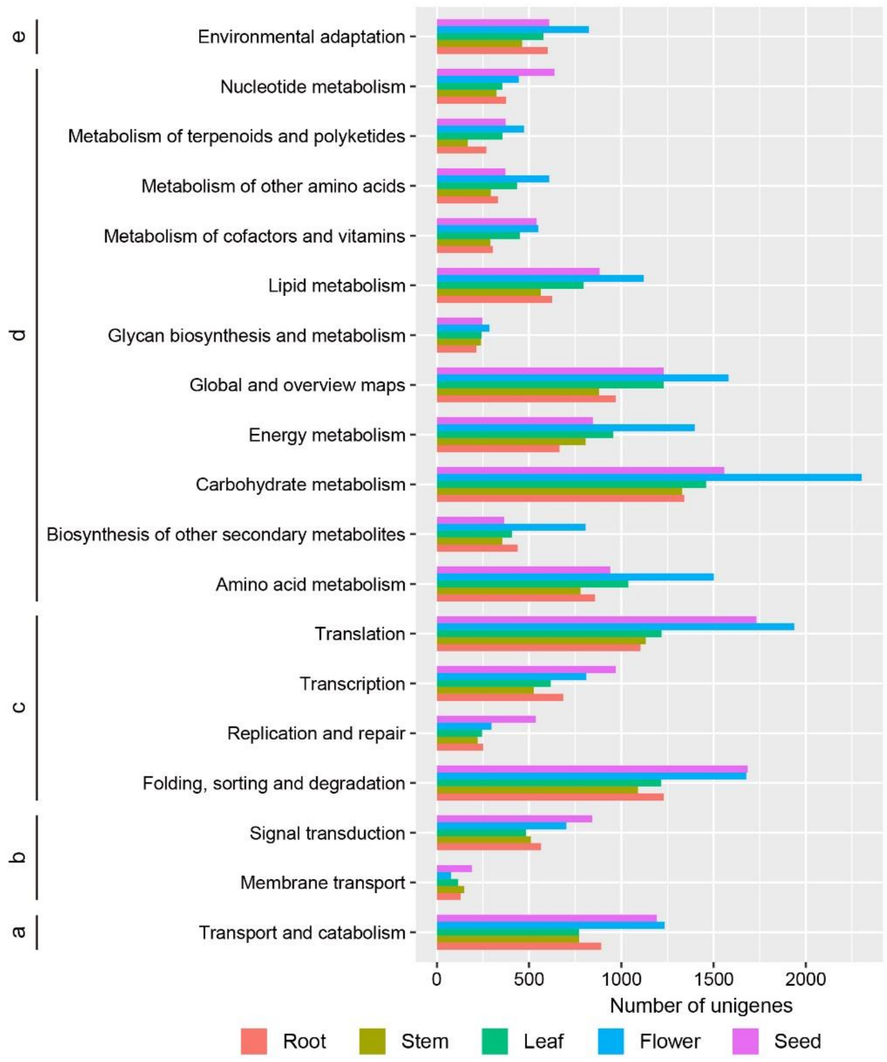

Figure S6. The Kyoto Encyclopedia of Genes and Genomes (KEGG) pathway annotation of the unigenes. a: cellular processes; b: environmental information processing; c: genetic information processing; d: metabolism; and e: organismal systems.

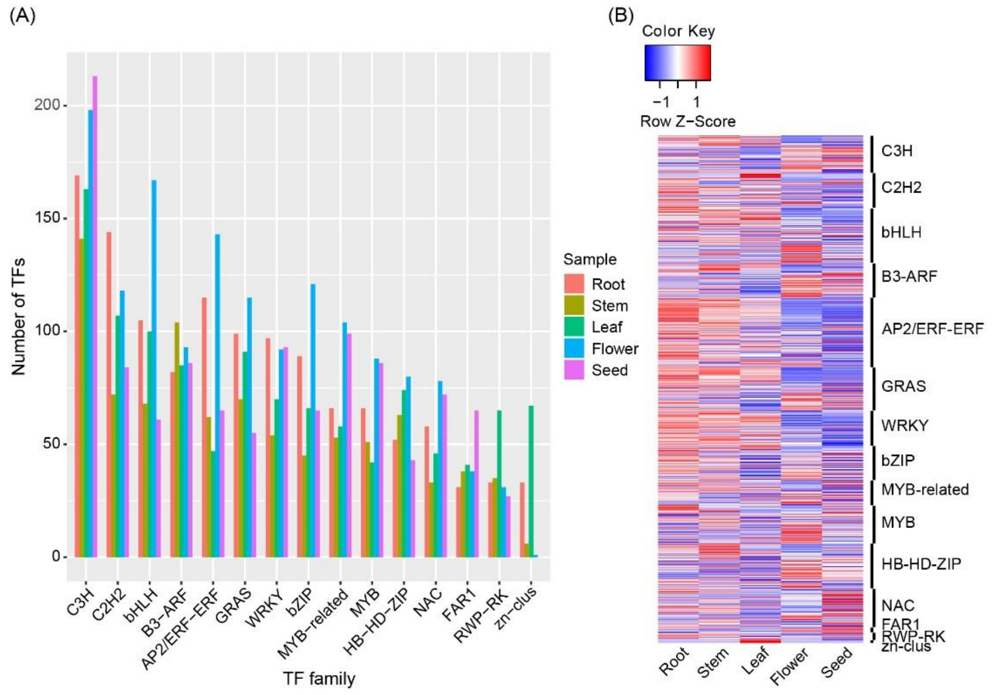

Figure S7. Identification of the transcription factors (TFs). **(A)** Distribution information of the top 15 TF families in the five organs of *M. micrantha*. **(B)** Expression heatmap of differentially expressed genes in the top 15 TF families. A scale indicates the color assigned to $\log_{10}$(FPKM + 1).

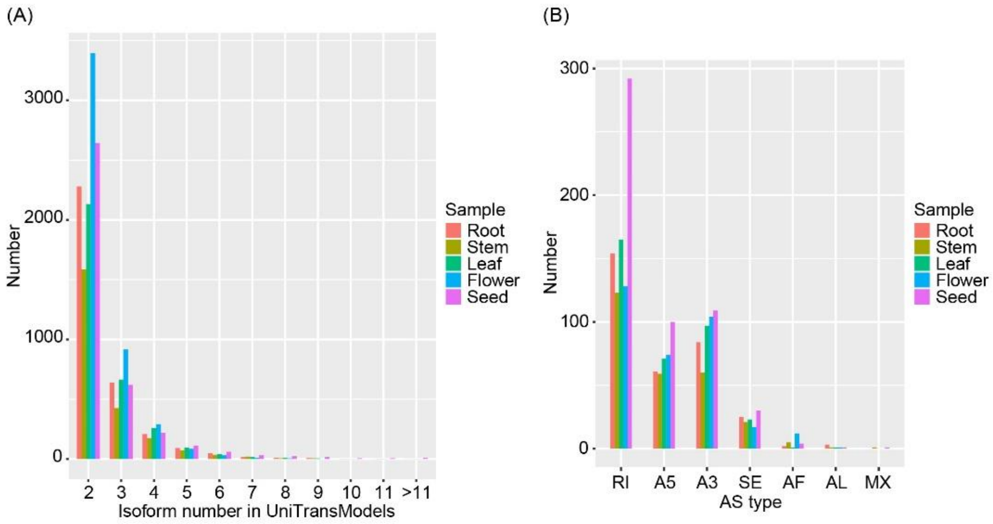

Figure S8. Identification of alternative splicing (AS) events in five organs. **(A)** Distribution of isoform numbers in UniTransModels. **(B)** The number of AS events in five organs.

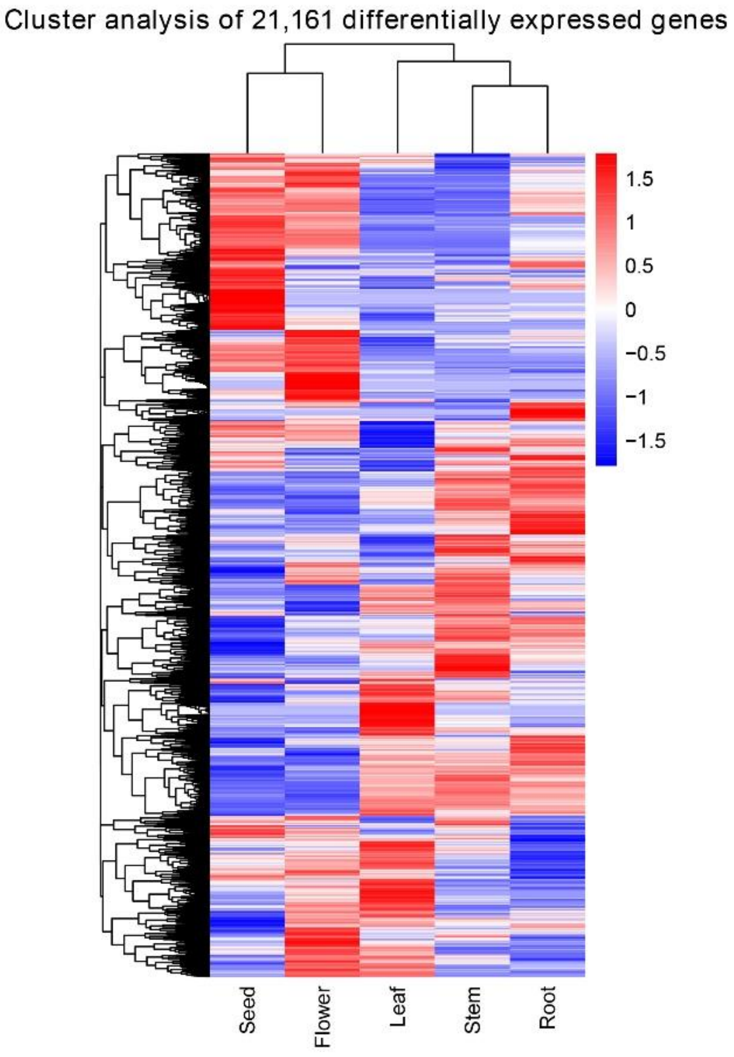

Figure S9. A heatmap of the expression patterns of 21,161 differentially expressed genes (DEGs) among five *M. micrantha* organs. A scale indicates the color assigned to $\log_{10}$(FPKM + 1).

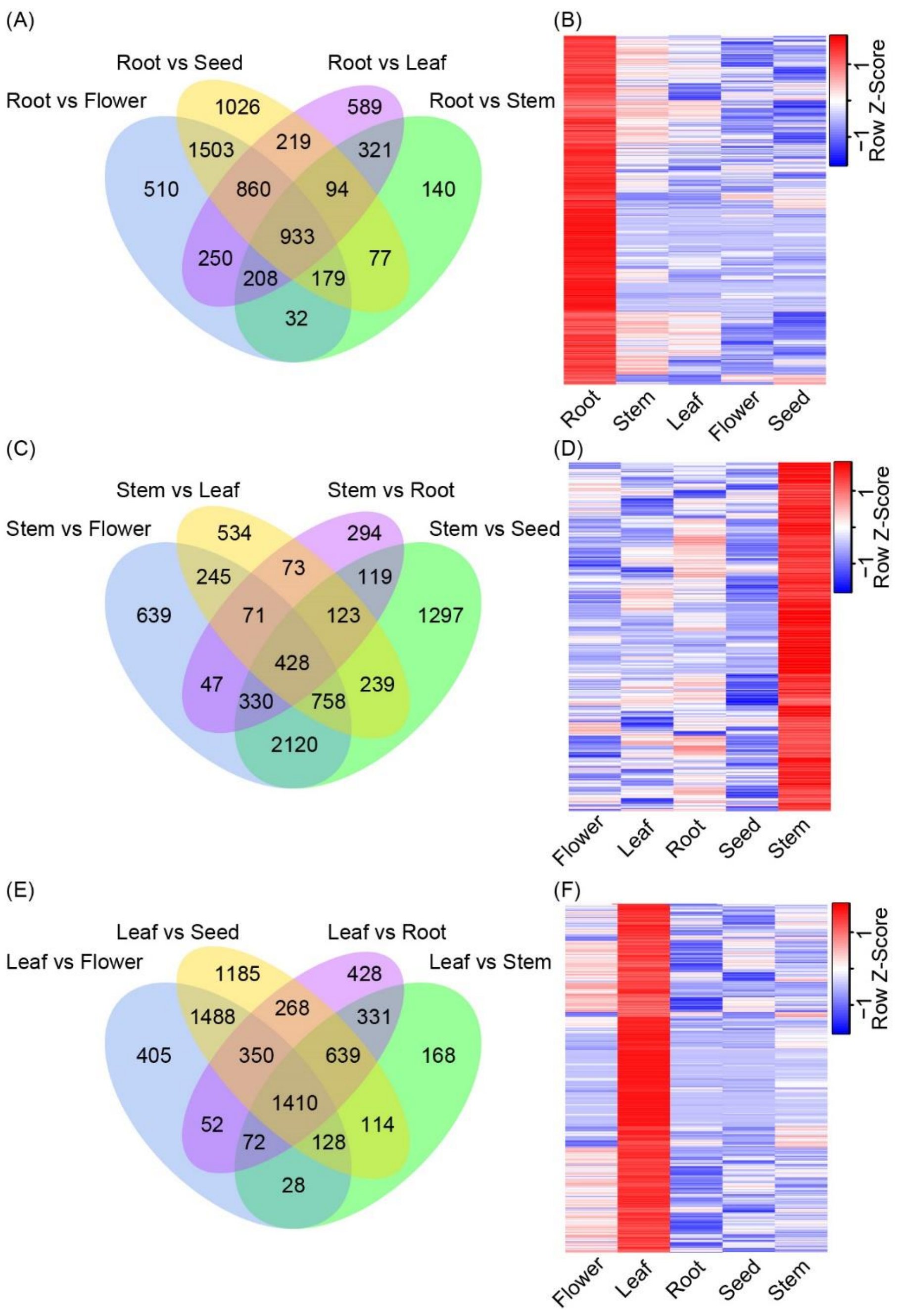

Figure S10. Venn diagram and heatmap of unigenes with higher expression in root, stem, and leaf. **(A)** Venn diagram of the number of unigenes with higher expression in root. **(B)** Expression heatmap of unigenes with higher expression in root. **(C)** Venn diagram of the number of unigenes with higher expression in stem. **(D)** Expression heatmap of unigenes with higher expression in stem. **(E)** Venn diagram of the number of unigenes with higher expression in leaf. **(F)** Expression heatmap of unigenes with higher expression in leaf. A scale indicates the color assigned to $\log_{10}$(FPKM + 1).

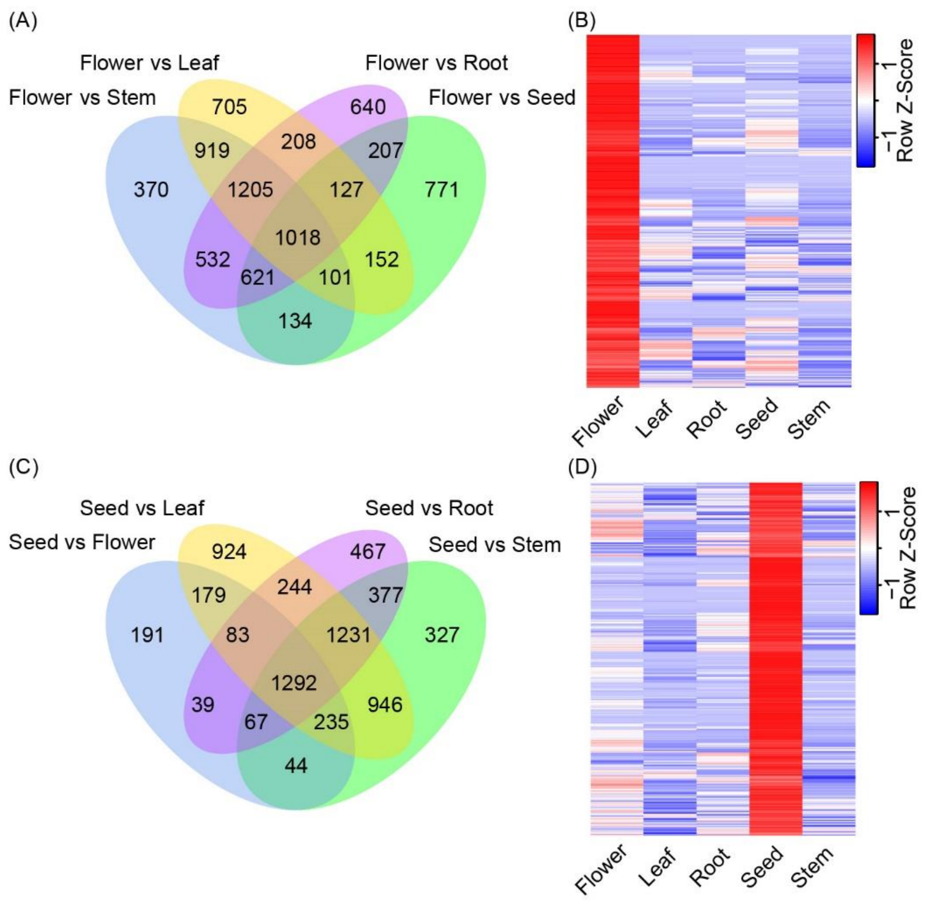

Figure S11. Venn diagram and heatmap of unigenes with higher expression in flower and seed. **(A)** Venn diagram of the number of unigenes with higher expression in flower. **(B)** Expression heatmap of unigenes with higher expression in flower. **(C)** Venn diagram of the number of unigenes with higher expression in seed. **(D)** Expression heatmap of unigenes with higher expression in seed. A scale indicates the color assigned to $\log_{10}$(FPKM + 1).

Supplementary Tables

Table S1. Summary of subreads from five *M. micrantha* transcriptomes generated by PacBio SMRT sequencing.

Sample	Library	Cell number	Subreads base (Gb)	Subreads number	Average subreads length (bp)	N50 (bp)
Root	1–2 kb	2	1.96	1,529,419	1282	1366
	2–3 kb	1	1.26	649,121	1939	2147
	3–6 kb	2	4.6	1,572,549	2924	3557
	All	5	7.82	3,751,089	2084	3040
Stem	1–2 kb	2	0.54	415,792	1306	1485
	2–3 kb	2	2.71	984,118	2751	3408
	3–6 kb	1	5.08	2,034,542	2499	3396
	All	5	8.33	3,434,452	2427	3367
Leaf	1–2 kb	1	0.38	245,077	1553	1732
	2–3 kb	1	5.7	2,658,396	2145	2567
	3–6 kb	1	1.99	996,707	1992	3470
	All	3	8.07	3,900,180	2069	2611
Flower	1–2 kb	2	2.55	2,158,018	1182	1488
	2–3 kb	1	5.11	2,900,351	1763	2268
	3–6 kb	1	5.2	3,476,650	1496	2250
	All	4	12.86	8,535,019	1508	2198
Seed	1–2 kb	1	1.35	1,152,462	1173	1432
	2–3 kb	1	1.22	863,750	1416	2216
	3–6 kb	2	9.25	2,419,634	3822	4706
	All	4	11.82	4,435,846	2665	4584

Table S2. Summary of *M. micrantha* transcripts generated by PacBio SMRT sequencing.

Sample	Number of CCS	Number of full-length transcripts	Number of FLNC transcripts	Average FLNC read length (bp)	Number of polished consensus reads	Average polished consensus read length (bp)	Number of unigenes	Average length of unigenes (bp)	N50 (bp)
Root	291,820	242,052	238,196	2633	92,242	2674	37,789	2821	3517
Stem	317,118	235,650	232,290	3070	77,761	3153	34,034	3148	3603
Leaf	309,347	214,261	211,535	2561	74,137	2568	38,100	2710	2973
Flower	452,378	263,692	257,905	1746	127,695	1627	54,937	1802	2238
Seed	304,786	236,511	231,877	3762	125,096	3646	53,906	3786	4707

CCS: circular consensus sequence; FLNC: full-length non-chimeric

Table S3. The length distribution of unigenes generated by Illumina and PacBio sequencing technology.

Sample	Sequencing platform	≤ 500 bp	500–1000 bp	1–2 kb	2–3 kb	3–4 kb	4–5 kb	> 5 kb	Total
Root	Illumina	24,163	48,767	39,139	9170	2204	544	246	12,4233
	PacBio	98	2492	9902	7273	11,068	5268	1688	37,789
Stem	Illumina	8475	18,997	22,090	7502	2293	591	284	60,232
	PacBio	318	1601	5332	5358	13,818	5719	1888	34,034
Leaf	Illumina	10,047	21,698	22,126	6871	1927	471	230	63,370
	PacBio	607	1231	5012	17,724	10,482	2237	807	38,100
Flower	Illumina	17,274	29,081	31,251	11,140	3137	861	484	93,228
	PacBio	3763	9442	19,595	15,502	5130	1041	464	54,937
Seed	Illumina	10,434	20,961	23,577	8100	2399	634	305	66,410
	PacBio	1475	3628	7582	4713	1710	23,095	11,703	53,906

Table S4. Summary of *M. micrantha* transcripts generated by Illumina RNA-Seq.

Sample	Number of raw reads	Number of clean reads	Clean bases (G)	Q20 (%)	Q30 (%)	Error (%)	GC (%)	Number of transcripts	Number of unigenes	Average unigene length (bp)	N50 (bp)
Root	44,881,058	43,232,272	6.48	97.07	92.75	0.02	45.67	253,652	124,233	1067	1336
Stem	42,965,818	40,269,280	6.04	96.72	91.53	0.02	43.58	91,842	60,232	1312	1685
Leaf	42,903,864	41,006,008	6.15	97.37	93.32	0.02	44.94	119,091	63,370	1226	1585
Flower	67,087,708	65,852,444	9.88	97.32	92.57	0.03	43.39	141,917	93,228	1250	1664
Seed	42,127,922	41,091,912	6.16	97.19	92.39	0.03	43.91	109,453	66,410	1286	1672

Table S5. The number of unigenes annotated to Gene Ontology (GO) terms under "response to stimulus" category in five *M. micrantha* organs.

GO ID	GO Term	Root	Stem	Leaf	Flower	Seed
GO: 0050896	response to stimulus	2509	2263	2384	2705	2993
GO: 0006950	response to stress	155	107	153	198	146
GO: 0006979	response to oxidative stress	102	83	51	104	76
GO: 0009607	response to biotic stimulus	34	26	26	35	50
GO: 0006952	defense response	60	43	71	90	78

Table S9. Statistics of the gene families related to biotic and abiotic factors for five *M. micrantha* organs.

Gene families	Root	Stem	Leaf	Flower	Seed
Terpene synthase (TPS)	62	26	59	37	12
Glutathione S-transferase (GST)	34	12	13	52	61

Table Captions

Table S6. Identification of transcription factors (TFs) in five organs of *M. micrantha*.

Table S7. Differentially expressed genes (DEGs) from paired comparisons among the five organs of *M. micrantha*.

Table S8. Kyoto Encyclopedia of Genes and Genomes (KEGG) enrichment analysis of unigenes with higher expression in each organ.

Table S10. Identification of the terpene synthase (TPS) gene family.

Table S11. Identification of the glutathione S-transferase (GST) gene family.

Table S12. Identification of the unigenes in the antioxidant defense system.

Table S13. Identification of the unigenes in the mevalonate (MVA) and methylerythritol phosphate (MEP) pathway.

Table S14. GenBank accession numbers of terpene synthase from other angiosperms.
